# Assessing athlete leadership and cohesion using a social network analysis approach

**DOI:** 10.3389/fpsyg.2023.1050385

**Published:** 2023-02-23

**Authors:** Ashley Flemington, Todd M. Loughead, Marie Desrosiers

**Affiliations:** Department of Kinesiology, Sport Psychology and Physical Activity Research Collaborative (SPPARC), University of Windsor, Windsor, ON, Canada

**Keywords:** athlete leadership, cohesion, group dynamics, social network analsysis, sport

## Abstract

The study of athlete leadership has gained momentum over the past 15 years and is recognized as a vital component of team performance. Specifically, athlete leadership has been most studied with regards to its impact on the outcome of cohesion. As a result, a current gap in this area of research is the analysis of attribute data, such as tenure and self-reported athlete leadership, and how this attribute data is related to outcomes, such as cohesion. However, much of current research examining this relationship has utilized traditional statistical methods, limiting interpretation of data because team members are inherently interdependent. One approach that considers the interdependence of team members is social network analysis (SNA). SNA facilitates the study of social structures within networks of people, such as a sports team, as well as individual attributes influencing or being influenced by the network. The present study used SNA to examine athlete leadership and cohesion within a sports team of 22 female professional hockey players. Participants self-reported tenure, completed a self-rated athlete leadership questionnaire, and rated each of their team members on network variables of athlete leadership and cohesion. The results showed that high network density and low degree centralization was found for both athlete leadership and cohesion networks, with high indegree centralities for each team member. Further, a strong correlation was found between the athlete leadership and cohesion networks (*p* < 0.001), indicating a positive relationship between the athlete leadership ties and the cohesion ties. Lastly, significant correlations were found between self-rated athlete leadership and the networks of athlete leadership and cohesion. Together these data suggest that a cohesive team shares leadership responsibilities with many ties between teammates.

## 1. Introduction

Athlete leadership is defined as “an athlete occupying a formal or informal role within a team who influences a group of team members to achieve a common goal” (Loughead et al., 2006, p. 144). This definition suggests that any athlete on a team can take on a leadership role. Thus, a team is not limited to a single athlete leader, but rather the leadership process is shared amongst several athletes depending on their leadership role. As noted in the above definition, athletes can occupy either a formal or informal leadership role. Athletes who occupy a formal leadership role are those that have been prescribed or selected into that position and are commonly known as captains, co-captains, or assistant captains (Loughead et al., 2006). In contrast, athletes who occupy an informal leadership role are those that ascend to their leadership role by being held in high esteem by their teammates despite not being formally recognized as a leader (Loughead et al., 2006). Having both formal and informal athlete leadership roles on a team enables several athletes to provide leadership to their teammates in a process known as shared leadership. As such, Loughead et al. (2021) advanced another definition to account for the shared nature of athlete leadership, describing it as “a shared dynamic team process composed of mutual influence and shared responsibility among team members, who lead each other toward the achievement of a common goal” (p. 161).

One reason athlete leadership is shared amongst numerous athletes is that there are multiple leadership behaviours to be performed thus, different team members can fulfil different behavioural roles. One set of leadership behaviours that athletes perform are transformational leadership behaviours. Within the context of athlete leadership, transformational leadership behaviours are those that consider the interest of teammates, assist teammates in being more aware of the importance of shared goals, and allow teammates to move beyond their own interests (Price and Weiss, 2013). The most utilized inventory to measure transformational leadership within athlete leadership research is the Differentiated Transformational Leadership Inventory (DTLI; Callow et al., 2009), which consist of six transformational leadership behaviours. The six dimensions include individualized consideration, inspirational motivation, intellectual stimulation, fostering the acceptance of group goals, high performance expectations, and appropriate role model. Individualized consideration assesses the leader’s personal attention to the follower and considers the follower’s individual needs. Inspirational motivation refers to a leader articulating a positive vision of the future and inspiring followers that they can achieve that vision. Intellectual stimulation is displayed when a leader challenges their followers to demonstrate creativity. Fostering acceptance of group goals refers to a leader promoting cohesion and cooperation by getting group members involved and committed to the group’s goals. High performance expectations occurs when a leader places high demands on the follower, expecting a high quality of work. Lastly, appropriate role model is displayed when a leader acts in ways that sets an example for followers.

While much of the research on leadership in the sport context focuses on coaches, the changing emphasis to athlete leaders has gained momentum as researchers have found that coaches and athletes exhibit different leadership behaviours (Loughead and Hardy, 2005). In their study, Loughead and Hardy (2005) found that coaches were perceived to exhibit more training and instruction, and autocratic decision-making behaviours. In contrast, athlete leaders were found to exhibit more social support, positive feedback, and democratic decision-making behaviours. As such, it is important to further our understanding of the role athlete leadership plays in team functioning and outcomes.

The current literature on athlete leadership has highlighted its importance as an integral component of successful team functioning (Loughead, 2017) with positive relationships being found between athlete leadership and athlete satisfaction (Paradis and Loughead, 2012), collective efficacy (Price and Weiss, 2011), and cohesion (Callow et al., 2009; Vincer and Loughead, 2010). As one of the most studied team outcomes, cohesion has been of particular interest as it relates to athlete leadership. More specifically, cohesion has historically been viewed as *the* most important small group variable (Lott and Lott, 1965; Carron et al., 1998), and as such is a construct that athlete leaders would want to foster on their teams since it is related to enhanced performance (Carron et al., 2002). For example, Vincer and Loughead (2010) conducted a study assessing athlete leadership and cohesion in varsity and club level athletes. Participants completed questionnaires assessing athlete leadership behaviours and cohesion on their team with the results showing that all four dimensions of cohesion were positively and strongly associated with each of the athlete leadership behaviours measured. The results from Vincer and Loughead assessed athlete leaders as a whole; meaning they did not discern between formal and informal athlete leaders. To address this limitation, Burkett et al. (2014) surveyed NCAA Division III basketball players regarding the formal and informal athlete leadership behaviours and cohesion on their team. The results of this study also found positive correlations between athlete leadership behaviours demonstrated by both formal and informal athlete leaders and dimensions of cohesion.

Researchers examining athlete leadership and cohesion often collect attribute data regarding characteristics such as tenure on the team; that is the amount of time the athlete has been a member of a particular team. This information is generally only used to describe the sample as a demographic variable, typically reported in terms of an average along with its standard deviation with the analysis not extending beyond this level. However, collecting these types of attribute data provides the opportunity to assess how factors such as tenure may impact the nature of athlete leadership and/or cohesion within teams. For instance, Fransen et al. (2015) used the demographic variables of competitive level and gender of the team to assess differences in perceived athlete leadership quality. In this study, no significant differences were found regarding athlete leadership quality based on team level (high vs. low), or gender. However, Duguay et al. (2019) also analyzed demographic data using SNA regression techniques (i.e., multiple regression quadratic assignment procedures), assessing relationships between leadership network and demographic data including age, playing position, leadership status, and nominations for the most skilled player on the team. It was found that skill nomination was a significant predictor of athlete leadership nomination for all teams, and being a formal leader (e.g., captain) was a significant predictor on two of the four teams sampled. Taken together, these studies provide evidence that the relationships between athlete leadership networks and demographic information is important to assess to better understand how these variables are related in various contexts.

In addition, relationships between attribute data of self-rated athlete leadership behaviour and network athlete leadership, as nominated by one’s teammates has not been previously examined. While researchers have explored the associations between different athlete leadership networks such as different athlete leadership roles (task, motivation, social, and external; Fransen et al., 2015), and dimensions of identity leadership (prototypicality, advancement, entrepreneurship, and impresarioship; Bruner et al., 2022), no current research has simultaneously examined self-reported athlete leadership and peer reported network athlete leadership. Assessing the difference between self-reported (attribute) and other-reported (network) leadership is of interest because previous literature has found discrepancies between these two types of information (e.g., Branson and Cornell, 2009; Yeatman and Trinitapoli, 2011), which may be due to factors such as social desirability bias (Gould, 1969) leading to over-estimating one’s own characteristics (An, 2022). Thus, discrepancies in research findings may become apparent as we transition from self-reported data to other-reported data when using Social Network Analysis (SNA). To address this shortcoming, the present study aims to assess relationships between self-rated leadership behaviours and other-rated network leadership using a SNA approach. This method is used to look at the interplay between attribute (self-rated) leadership of individual team members and the leadership relations between teammates that has been previously studied separately (e.g., Callow et al., 2009; Loughead et al., 2016). To further extend this area within athlete leadership research, using both self-rated and other-rated leadership allows both of these measures to be assessed with network cohesion, providing a more in-depth investigation into the athlete leadership – cohesion relationship.

Although the majority of research supports a positive relationship between athlete leadership and cohesion, much of this research has been done using traditional statistical techniques. While this is common practice, and necessary for certain research questions, traditional statistics such as null hypothesis significance testing like t-tests, ANOVAs, and regressions, have a limitation when it comes to research on social groups, such as sport teams (e.g., Prell, 2012; Borgatti et al., 2013; Duguay et al., 2020). The use of null hypothesis significance testing requires that certain assumptions about the data are met for the test to be reliable. For example, these types of tests require the data to be independent, meaning each rating is not related or correlated to another rating. In research on social groups, we know this assumption is impossible to satisfy since members of social groups are inherently interdependent, thus the data collected on the social relationship between members is inherently interdependent as well. SNA techniques are a way to overcome this issue of data dependency. SNA is a group of methodologies and statistical techniques used for the study of social groups by assessing social structures within a network of people (Borgatti et al., 2013). These techniques allow for the assessment of relational ties between members of a social group, as well as examining how individual attributes of the members influence, or are influenced by, the network as a whole. While SNA originated from fields such as sociology and social psychology, SNA is useful in sport psychology as the inherent dependency of data within a team analysis is overcome. Significance testing with SNA techniques uses permutation tests in which data are reordered and reanalyzed many times creating a distribution of potential outcomes for the given data. This distribution, created through many permutation tests, is then used to compare the data for determining the significance levels of the results (Prell, 2012).

Given the advantage of overcoming the issue of data dependency, it is not surprising that there has been an increasing prevalence of SNA techniques to examine team-based relationships and outcomes in sport psychology (e.g., Fransen et al., 2015; Duguay et al., 2019). While research in this area is still in its infancy and there is not a wide breath of literature on many team outcomes, cohesion is one team outcome that has garnered some research attention. For example, Loughead et al. (2016) used SNA techniques to examine the relationship between athlete leadership quality and cohesion, finding a positive correlation between these two networks. Loughead et al. (2016) also examined the association between four athlete leadership roles and cohesion, also finding positive correlations between these networks.

Within SNA research there are multiple design types, two of the most relevant for studying sports teams are whole networks and multiple networks (Robins, 2015). A whole network design consists of a full set of actors within a well-defined network boundary, for example all (or most) of the athletes (i.e., actors) of a single sport team (e.g., Passos et al., 2011). In contrast, the multiple network design consists of more than one whole network, for example members coming from two or more teams (e.g., Fransen et al., 2017) where the teams’ data are generally aggregated for analyses. The networks (e.g., sport teams) in a multiple network design need to be different from one another such that there is not social overlap between the networks. Consequently, this assumption can be challenging to achieve within a multiple network design (Robins, 2015). Further, while multiple network designs allow for a greater generalization, detail and specificity within a team is lost, limiting the specific conclusions that can be drawn. With whole network designs a high response rate is needed from all (or most) group members (e.g., athletes from one team), as it allows conclusions to be drawn about the entire social system of a given network (e.g., one sport team).

Taken together, the purpose of the current study was to advance our understanding of tenure and self-rated leadership and how they relate to cohesion and athlete leadership using a whole network design. To achieve our objectives, we assessed the network density and centralization as well as degree centrality for both the cohesion and athlete leadership networks. As well, associations between the athlete leadership and cohesion networks were assessed, and finally associations between attribute data (tenure, and self-rated leadership) and both networks were assessed using data from one sport team.

## 2. Method

### 2.1. Participants

Participants included members of one professional female ice hockey team from a North American league. Of the 22 members who were rostered on the team, 19 of them agreed to participate in the current study. The athletes ranged in age from 22 to 30 years old (*M* = 24.63 ± 2.48) with team members having played on this team between 1 to 5 years (*M* = 2.32 ± 1.49).

### 2.2. Measures

#### 2.2.1. Demographics

Demographic information collected included age, tenure on the team, and playing position (right wing, center, left wing, right defence, left defence, or goalie).

#### 2.2.2. Self-rated leadership

In order to assess self-rated leadership, each participant completed 23 items from the Differentiated Transformational Leadership Inventory (DTLI; Callow et al., 2009) that is scored on a 5-point Likert scale from 1 (*not at all*) to 5 (*all of the time*) to measure six dimensions of transformational leadership. These dimensions include individual consideration, inspirational motivation, intellectual stimulation, fostering acceptance of group goals and promoting teamwork, high performance expectations, and appropriate role model. An overall transformational leadership score was calculated for each participant by summing the scores of the six dimensions.

#### 2.2.3. Network athlete leadership

To measure athlete leadership at the network level, athletes were asked to rate the leadership effectiveness for each of their teammates. A one item statement was used for each of the six dimensions of the DTLI and were scored on a scale from 1 (*very poorly*) to 5 (*very well*). These one-item statements were derived from the definitions of each of the DTLI dimensions. For inspirational motivation, participants were asked “How well does each member of your team energize you by presenting an optimistic view of the future concerning the team’s goals?.” For appropriate role model, participants were asked “How well does each member of your team serve as a role model for you?.” For fostering acceptance of group goals, participants were asked “How well does each member of you team cooperate with you in working towards the team’s goals?.” For high performance expectations, participants were asked “How well does each member of your team stress the importance of striving for excellence by having high personal performance standards?” For intellectual stimulation, participants were asked “How well does each member of your team challenge you to view problems from different perspectives.” For individualized consideration, participants were asked “How well does each member of your team show an interest in your own development as a player on this team?” An overall score for network athlete leadership for each participant was calculated by calculating the sum of these six dimensions.

#### 2.2.4. Network cohesion

To measure cohesion at the network level, athletes were asked to rate how cohesive they felt with each of their teammates. Items were based on Carron et al. (1985)’s framework distinguishing between task and social components. One item was used to assess the social component of cohesion, referring to the development and maintenance of social relationships, asking participants to “Please indicate the extent to which you feel united with each of your teammates in order to maintain good social relationships within the team.” One item was also used to assess the task component of cohesion, referring to the achievement of group goals and objectives (Carron et al., 1985) asking participants to “Please indicate the extent to which you feel united with each of your teammates in order to achieve the team’s goals and objectives.” Athletes rated these items on a scale from 1 (*Not united at all*) to 5 (*Extremely united*). These one-item statements were derived from the definitions of each form of cohesion.

### 2.3. Procedure

Following ethics clearance from the university’s Research Ethics Board, four female professional hockey teams from a North American league were contacted through email regarding their interest to participate in the study. We received a response from one general manager indicating a desire to participate in the study. Consequently, a second email was sent to the general manager who then forwarded it to the athletes on the team that provided a description of the study. Those athletes interested in participating were instructed to email the researcher, who then sent an email containing a link to the study’s survey. Upon opening the study’s survey link, participants were first asked to provide informed consent and once this was completed, they were directed to the survey. The survey took approximately 30 min to complete.

### 2.4. Data analysis

All analyses on the social network data were conducted using version 6 of the UCINET software (Borgatti, 2002) and visualizations utilized the NetDraw tool within this software program (Borgatti et al., 2002). At the network level of analysis, density and degree centralization were calculated for the athlete leadership network and both task and social cohesion networks. First, density measures the proportion of possible ties in a given network that are present in the whole network. We calculated valued (data with more numerical value options than one and two) and directional data (data values that go from one athlete to another, and the reciprocal data does not need to match) for this analysis. For the present study, the density for any given network can range from 1 (low density) to 5 (very high density). Second, degree centralization assesses the extent to which any one athlete receives all of the ties within the network. Thus, a high degree of centralization would indicate that the network density is focused on a single athlete. For the present study normalized degree centralization scores were used such that scores could range between 0 (no centralization) to 1 (completely centralized on a single athlete). At the individual athlete level of analysis, indegree centrality was calculated for the athlete leadership and cohesion networks. This assesses the number of ties received by an athlete from their teammates indicating their involvement in the network (Prell, 2012), thus a high indegree centrality for a given athlete suggests many teammates nominate them. Data were dichotomized for this analysis, providing a binary and directional network, thus scores could range from 0 to 1.

Lastly, permutation tests were used to assess correlations between the athlete leadership and cohesion networks, as well as between attribute data and each of the networks. To assess the relationship between the athlete leadership and each of the two cohesion networks a Quadratic Assignment Procedure (QAP) correlation was computed. This provides a Pearson’s *r* correlation between the two networks, determining if the presence of a tie between members in one network corresponds to a tie between those same members in the another network. Further, to assess the relationship between attribute data (tenure, and self-rated athlete leadership) and each of the networks (i.e., athlete leadership, task cohesion, social cohesion), Moran’s autocorrelation techniques were used. This technique allows for discrete or continuous variables, such as attribute data, to be correlated with the network data. Interpreted similarly to a Pearson’s *r*, a larger positive Moran’s *I* is indicative of a greater positive autocorrelation (Borgatti et al., 2002). QAP correlations and autocorrelations are most appropriate for these data, as they are non-parametric tests using permutations to determine significance, thus they are more robust to violations of the assumption of independence (Krackhardt, 1988) compared to traditional significance testing techniques.

## 3. Results

**Figure 1:**
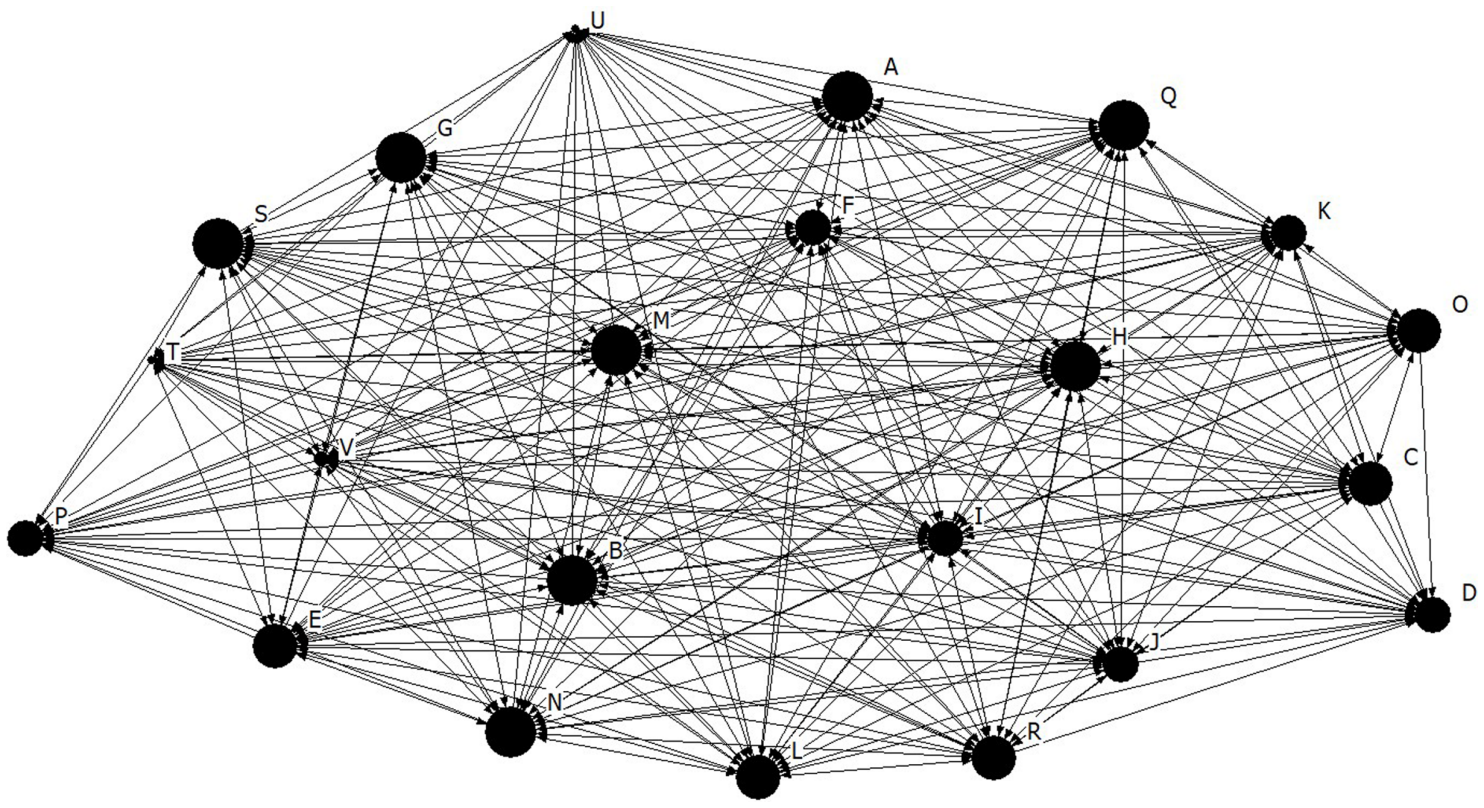
Network diagram for athlete leadership. Dots indicate each team member, Size of dot indicates relative indegree centrality for athlete leadership.

**Figure 2:**
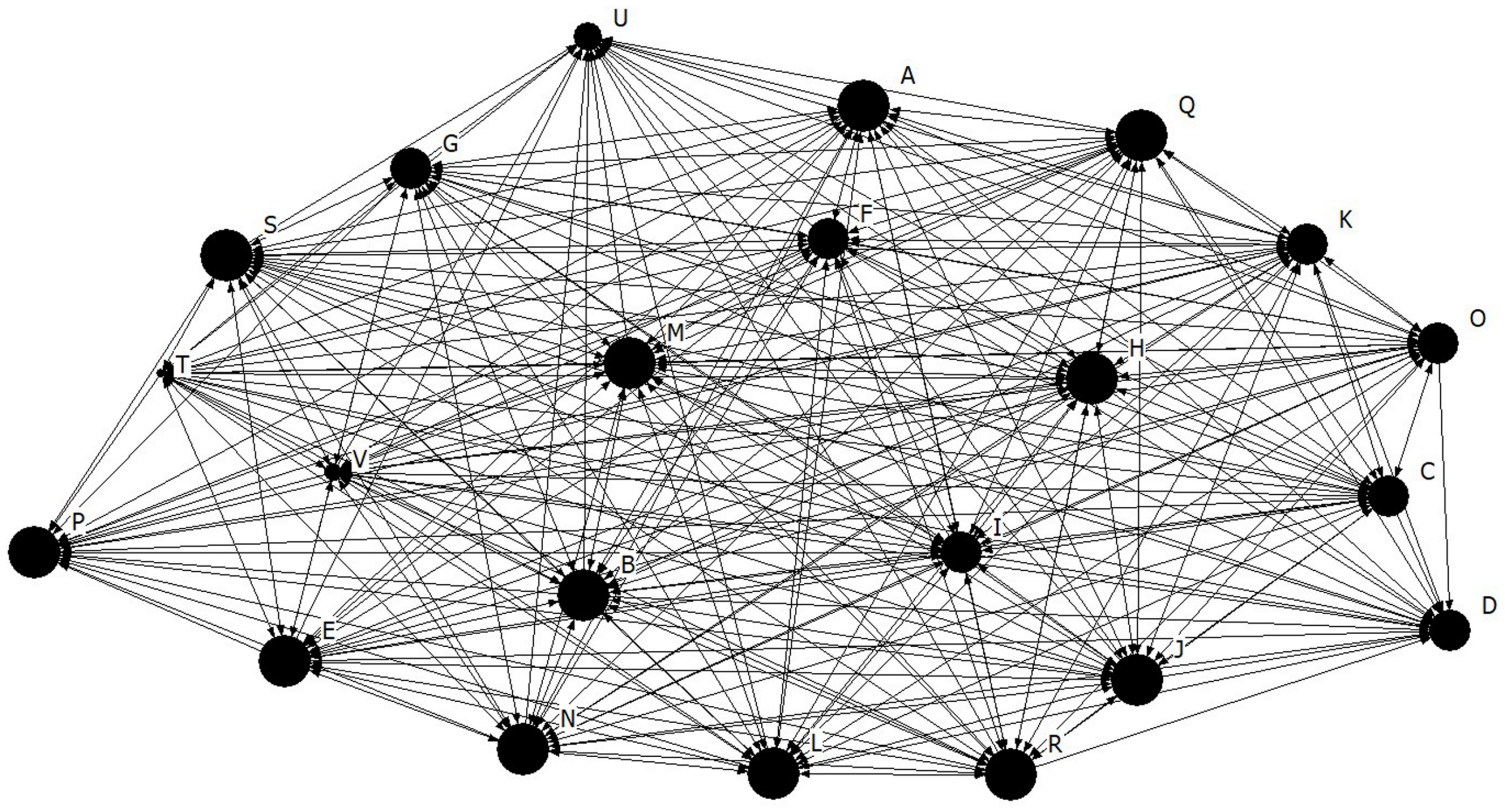
Network diagram for task cohesion. Dots indicate each team member, Size of dot indicates relative indegree centrality for task cohesion.

**Figure 3:**
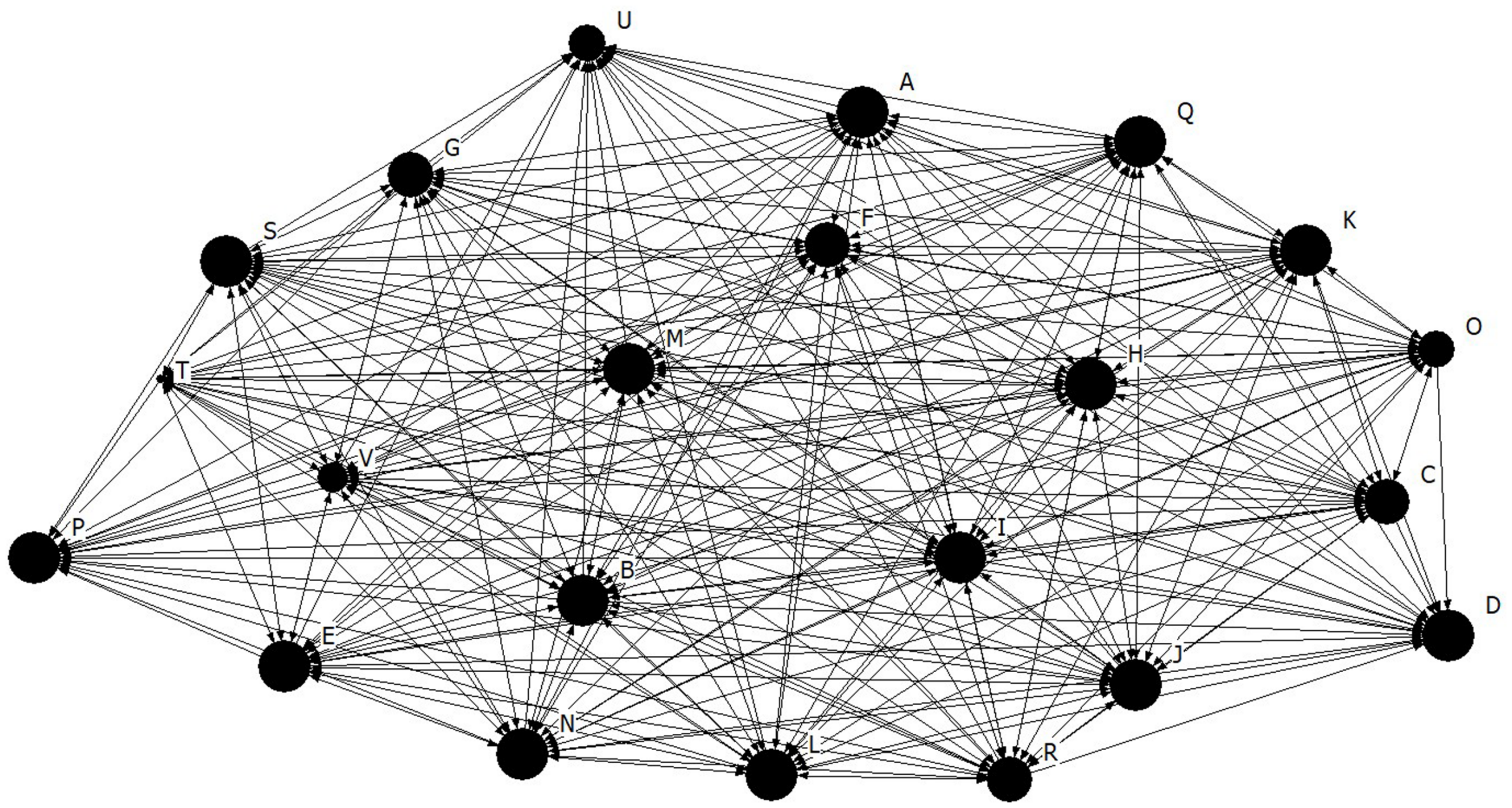
Network diagram for social cohesion. Dots indicate each team member, Size of dot indicates relative indegree centrality for social cohesion.

Density for the athlete leadership network was found to be high at 3.98 ± 0.80, out of a possible 5 (Figure 1). The density for both the task (4.15 ± 0.89) and social (4.11 ± 0.92) cohesion networks were also high, and relatively higher than the athlete leadership network for this team (Figures 2, 3, respectively). These findings suggests that there are many connections between teammates regarding athlete leadership, social cohesion, and task cohesion.

Centralization for the team was found to be low, with a normalized indegree centralization of 0.08 for the AL network and 0.04 for both the task and social cohesion networks. With the network level indegree centralization being low, these results suggests that a single athlete is not receiving all of the connections, rather these relationships are shared among many of the athletes. The indegree centralities of individual athletes for all networks were found to be high and are reported in Table 1, indicating that many athletes are perceived to be providing these leadership and cohesion relationships to their teammates.

**Table 1:** Normalized indegree centrality for Athlete Leadership and Cohesion networks.

Athlete	Athlete leadership	Task cohesion	Social cohesion
A	0.86	0.86	0.86
B	0.86	0.86	0.86
C	0.81	0.81	0.81
D	0.76	0.81	0.86
E	0.81	0.86	0.86
F	0.76	0.81	0.81
G	0.86	0.81	0.81
H	0.86	0.86	0.86
I	0.76	0.81	0.86
J	0.76	0.86	0.86
K	0.76	0.81	0.86
L	0.81	0.86	0.86
M	0.86	0.86	0.86
N	0.86	0.86	0.86
O	0.81	0.81	0.76
P	0.76	0.86	0.86
Q	0.86	0.86	0.86
R	0.81	0.86	0.81
S	0.86	0.86	0.86
T	0.57	0.67	0.57
U	0.57	0.76	0.76
V	0.62	0.71	0.71

The results also showed a large and significant positive correlation between the athlete leadership and task cohesion networks (*r* = 0.71, *p* < 0.001) as well as between the athlete leadership and social cohesion networks (*r* = 0.57, *p* < 0.001). These findings indicate that there is a strong positive relationship between these networks, highlighting that when a relationship exists in one network (i.e., leadership), it is most likely to exist in another network (i.e., social cohesion).

Correlations between attribute data and each of the networks were also computed. For the athlete leadership network non-significant relationships were found for tenure (*I* = −0.53, *p* = 0.29), suggesting number of years on this team is not indicative of being perceived as a leader. Importantly, a significant and positive correlation was found between the athlete leadership network and self-reported athlete leadership behaviours (*I* = 0.003, *p* < 0.001), meaning those athletes who rated themselves as leaders, were also viewed as leaders by their teammates.

For the cohesion networks, non-significant relationships were also found for tenure (task: *I* = −0.05, *p* = 0.33; social: *I* = −0.05, *p* = 0.38), again indicating that number of years on the team is not associated with perceptions of cohesion. Interestingly, a significant and negative correlation was found between the cohesion network and self-reported athlete leadership behaviours (task: *I* = −0.001, *p* < 0.001; social: *I* = −0.005, *p* < 0.001), which indicates that those who rated themselves as leaders were perceived as being less cohesive by their teammates.

## 4. Discussion

The purpose of the present study was to investigate cohesion and athlete leadership using SNA, and to further our understanding of how tenure and self-rated leadership are related to these networks. First, high density and individual centralities coupled with low centralization suggests that the team from the present study had many well dispersed connections for both their athlete leadership and cohesion relationships. According to Prell (2012) high density alone is not enough to indicate a well-connected group because those connections could be occurring through a single or small group of key members. Thus, the high density found within the current team is best interpreted along side the low centralization score, which supports the idea that connections are not concentrated on a few team members, rather they are spread well throughout the team. Many well dispersed athlete leadership connections suggests that leadership responsibilities were shared amongst a large portion of the team. These results mirror those of Duguay et al. (2019) who also found that all soccer players were nominated by at least one teammate as being a leader, therefore demonstrating the presence of shared athlete leadership. Similarly, many well dispersed cohesion connections suggest that all members of the team get along well with each other, feeling connected and united toward team goals. This overall pattern of results, where a sports team has a high density coupled with a low centralization, has been seen in previous literature matching the visual interpretation of the networks sociogram supporting a shared nature of athlete leadership (Duguay et al., 2020). Importantly, this pattern of results further supports the notion that a formal leadership role occupied by a single athlete, such as a captain, does not fulfill the entire athlete leadership role within a team (Fransen et al., 2014). The results of the present study are substantiated by Fransen et al. (2014) who found that 43.6% of participants did not report their captain as their strongest leader. Taken together, the results of previous studies along with the findings of the present study, provide empirical support of shared athlete leadership as many athletes on a team take part in a process of mutual influence and shared responsibilities (Loughead et al., 2021).

Further, the associations between network athlete leadership and each of the cohesion networks were also assessed and found to be positive. This means there was a high degree of agreement between the networks, when a connection existed in the athlete leadership network, it was also highly likely to exist in the cohesion networks. Practically speaking, athletes that were rated highly on their leadership relationships were also rated highly on their cohesion relationships. As previously mentioned, this relationship is well established in previous literature using traditional statistical methods (e.g., Callow et al., 2009; Vincer and Loughead, 2010) with a similar pattern emerging when using social network analysis techniques. The only other study to our knowledge to assess athlete leadership and cohesion using SNA is Loughead et al. (2016), who found that, for most teams in their study, both social and task cohesion were positively and significantly correlated to four leadership dimensions (task, motivational, social, and external). Thus, the present study provides further support for the importance of the athlete leadership – cohesion relationship using SNA and expands upon this by utilizing the six dimensions of the DTLI for assessing leadership behaviour. This provides support for the association existing not only with the four types of leadership, but also six transformational leadership behaviours, which highlights the complexity of this relationship whereby numerous athlete leadership behaviours are related to task and social cohesion.

The relationship between athlete leadership and cohesion is important for sport teams because both athlete leadership and cohesion are positively associated with performance (Carron et al., 2002; Fransen et al., 2017). The direction of these relationships are reciprocal, such that cohesion improves performance and good performance improves cohesion (Carron et al., 2002). This is also the case for the relationship between leadership and performance, as indicated by the Multidimensional Model of Leadership (Chelladurai, 2007), where leadership characteristics positively impact leadership behaviour which in turn improves performance, and improved performance enhances positive leadership behaviours. Taken together, there are many positive relationships between leadership, cohesion, and performance, and thus are important for improving team effectiveness.

Lastly, the present study assessed relationships between attribute data (tenure and self-reported leadership) and each of the networks (cohesion and athlete leadership). Tenure was not found to be associated with any of the networks, suggesting that regardless of how long a team member has played for the team they provided and received leadership, and experienced similar feelings of cohesion with their teammates. This was an interesting finding as it suggests strong intra-team relationships, however it contradicts some previous research findings that suggests higher tenured athletes are perceived as providing more leadership. For example, Loughead et al. (2006) found that between 70 and 88% of intercollegiate athletes classified as a leader were in their third or fourth year on the team. Similarly, Duguay et al. (2018) found that intercollegiate athletes placed greater importance on higher tenured teammates (years four and five on the team) to show leadership compared to lower tenured teammates (years two and three on the team). Similarly, greater importance for showing leadership was placed on those athletes in their second and third year, compared to those in their first year on the team. There are a few different explanations for these differing findings. First, the present study may show a different relationship due to how highly dense and cohesive this particular team was; thus, the density of the team may act as a moderating factor for the relationship between an athlete leadership or cohesion network and athlete tenure. Second, the current study sampled athletes playing at a professional level compared to studies (Loughead et al., 2006; Duguay et al., 2018) where the athletes were competing at an intercollegiate level. At the intercollegiate level, coaches place a major emphasis on developing their athlete leaders gradually over the course of their five-year career (Duguay et al., 2020). In contrast, it may be the case that athletes at the professional level are expected to provide leadership as soon as they join the team. Future research should examine whether and what are the different expectations for leadership based on playing level.

Interestingly, while self-reported athlete leadership was positively associated with the athlete leadership network, it was negatively associated with both cohesion networks. Meaning that individuals that rate themselves highly as leaders, are also perceived highly as leaders within the team, however these individuals tend to be rated lower on being cohesive with their teammates. This is an unexpected finding since typically athlete leadership and cohesion are highly correlated, however this is the first study to assess both self-rated athlete leadership and network athlete leadership simultaneously. These results suggest that some differences may exist between one’s self-perception of leadership and teammates perception of their leadership as it relates to cohesion between members, and further investigation into this discrepancy is warranted.

Along with the many strengths and insights of this study, there remains limitations to be considered during interpretation of the results and for future research in this area. Firstly, looking at a single team strengthens our ability to assess and understand that team but limits the generalizability to any other context. As such future research should conduct multiple whole network analyses without aggregating the data, where several teams of equal sizes are analyzed independently but also allowing for comparisons between teams broadening the scope of the research. To expand further on this area of research, it will also be important to examine teams with various amounts of athlete leadership density, including densities lower than the team in the present study. Assessing this diversity may provide insight into how variations in athlete leadership relationships and the sharedness of athlete leadership influences associations with cohesion and attribute data. Secondly, the present study was a cross sectional design, as such changes in the teams’ relationships across the season as well as potential casual relationships are not captured in the present data. Moving forward, research should aim to collect data from teams across multiple time points of a season, to better capture the changes and development of team relationships. Lastly, due to the nature of data collection for SNA studies and participant burden, the full DTLI could not be used for network athlete leadership. As such each dimension of the DTLI was collapsed into a single representative item, reducing the items to six as opposed to the original 23. As a result, these specific six items have not been previously validated. Bruner et al. (2022) recommended this approach when conducting SNA.

The present study used both visual and quantitative SNA techniques to assess the team’s athlete leadership and cohesion relationships as well as the relationships between attribute data and the networks. Overall, the present study found a highly cohesive team that shares leadership responsibilities across many members, regardless of one’s tenure on the team.

## Data availability statement

The raw data supporting the conclusions of this article will be made available by the authors, without undue reservation.

## Ethics statement

The studies involving human participants were reviewed and approved by Research Ethics Board, University of Windsor. The patients/participants provided their written informed consent to participate in this study.

## Author contributions

MD completed participant recruitment and data collection and aided in the study conceptualization. TL aided in the study conceptualization and development, as well as writing the manuscript. AF completed the data cleaning and analysis and was the primary author of the manuscript. All authors contributed to the article and approved the submitted version.

## Conflict of interest

The authors declare that the research was conducted in the absence of any commercial or financial relationships that could be construed as a potential conflict of interest.

## Publisher’s note

All claims expressed in this article are solely those of the authors and do not necessarily represent those of their affiliated organizations, or those of the publisher, the editors and the reviewers. Any product that may be evaluated in this article, or claim that may be made by its manufacturer, is not guaranteed or endorsed by the publisher.
